# Distinct oral DNA viral signatures in rheumatoid arthritis: a Pilot study

**DOI:** 10.1080/20002297.2024.2348260

**Published:** 2024-05-01

**Authors:** Mahin Ghorbani, Nooshin Khoshdoozmasouleh

**Affiliations:** a Department of Dental Medicine, Karolinska Institute, Stockholm, Sweden; b Department of Laboratory Medicine, Karolinska Institute, Stockholm, Sweden; c Department of Molecular Medicine, University of Padova, Padova, Italy; d Roswell Park Comprehensive Cancer Center, Department of Cancer Genomics, Buffalo, NY, USA

**Keywords:** Oral virome, rheumatoid arthritis (RA), whole genome sequence (WGS), herpesviruses, Bacteriophage, Herpesvirus

## Abstract

**Background:**

Despite evidence linking viruses and oral microbiome to rheumatoid arthritis (RA), limited whole genome sequencing research has been conducted on the oral virome (a viral component of the microbiome) of untreated RA patients. This pilot research seeks to address this knowledge gap by comparing the oral virome of untreated rheumatoid arthritis patients (RAs) and healthy individuals (HCs).

**Method:**

Whole genome DNA sequence of saliva samples from 45 participants including 21 RAs and 24 age and gender matched HCs was obtained from the BioProject: PRJEB6997. Metaphlan3 pipeline and LEfSe analysis were used for the viral signature detection. Wilcoxon pairwise test and ROC analysis were used to validate and predict signatures.

**Results:**

RA exhibits higher alpha diversity compared to HCs. *Callitrichine gammaherpesvirus* 3, *Human gammaherpesvirus* 4 (EBV), *Murid betaherpesvirus* 8, and *Suid alphaherpesvirus* 1 were enriched in RAs, while Aotine betaherpesvirus 1 from the Cytomegalovirus genus was enriched in HCs. In addition, *Saccharomyces cerevisiae* killer virus M1 (ScV-M1) was found to be enriched in RAs, whereas bacteriophage Hk97virus (Siphoviridae) and Cd119virus (Myoviridae) were enriched in HCs.

**Conclusion:**

This study identifies significant DNA oral viral signatures at species level as potential biomarkers for the early detection and diagnosis of rheumatoid arthritis.

## Background

Rheumatoid arthritis (RA) is a predominant autoimmune inflammatory condition [1], the disease is characterized by synovial joint inflammation, which leads to cartilage, bone, and tendon destruction [2]. The disease can be diagnosed using autoantibodies such as rheumatoid factor (RF) and anti-citrullinated peptide antibodies (ACPAs) [3]. An extensive meta-analysis, encompassing 67 studies conducted from 1980 to 2019, aimed to ascertain the worldwide prevalence of rheumatoid arthritis. The study of 742,246 people with rheumatoid arthritis (RA) and 211,592,925 without RA found a global prevalence of 0.46% (95% confidence interval 0.39–0.54; I2 = 99.9%) [4]. Various genetic and environmental factors have been associated with the onset of RA, even though the precise etiology of the condition remains elusive [5]. The HLA-DRB1 locus is among the most significant genetic risk factors for RA and ACPAs. It is estimated that 80% of ACPA+ RA patients contain alleles encoding for a five-amino acid sequence known as the common epitope in the HLA-DRB1 region, which correlates with disease activity and mortality. Yet, not all carriers of RA susceptibility alleles acquire the disease [6,7].

Several environmental factors, including exposure to tobacco smoke, air pollution, high salt, and red meat diet, obesity, and inadequate vitamin D intake, are related to an increased risk of RA [5,8–11]. Despite the growing array of treatment options, healthcare providers still face challenges in tailoring effective treatments for individual patients with RA. In accordance with the concept of treat to target, rheumatologists frequently cycle or switch drugs to achieve stringent disease management [12,13]. Owing to the intricate nature of disease management, a considerable number of patients experience non-responsiveness to multiple biological disease-modifying antirheumatic drugs (bDMARD) in real-world clinical settings [14,15]. Notably, a substantial portion, approximately two-thirds, exhibit inadequate response to the initial tumor necrosis factor inhibitor (TFNI) within 6 months of treatment, with at least 12% discontinuing the second bDMARD due to inefficacy [15]. Consequently, it becomes imperative to delve into alternative diagnostics, therapies, and potential biomarkers to address the elusive facets of RA. Recent studies on RA highlight the microbiome as a crucial focus for creating new diagnostic and treatment methods, which could lead to targeted interventions and deepen our understanding of this intricate autoimmune condition [16,17].

The human virome is a diverse collection of viruses that exist inside or on the human body, consisting of around 10^ [13] particles per person [18]. The virome is composed of a diverse range of eukaryotic viruses and prokaryotic viruses including bacteriophages [18,19]. Eukaryotic viruses exhibit a wide range of effects on human health, varying from mild, self-restricting acute or chronic infections to severe pathological consequences [19]. Bacteriophages, which make up the majority of human virome, have a crucial function in intricately controlling bacterial ecosystems. Their role is centered around maintaining the fragile balance of bacteriome diversity and abundance. The imbalance in the bacteriome, which is affected by the dynamic interactions between viruses and bacteria, has the capacity to cause many clinical disorders, such as RA [19–21]. The role of viruses in the complex landscape of RA development is viewed as dual, involving active participation in disease pathogenesis while also acting as a preventative measure against bone degradation, indicating a nuanced link between the virome and the pathophysiology of RA [22].

One well-studied member of the *Herpesvirus* family is the *Epstein Barr virus (EBV)*, which has long been hypothesized to play a role in the etiology of RA [23]. Alspaugh, M A *et al*. revealed that the serum of RA patients included significant levels of antibodies against a nuclear antigen present in EBV-infected cells. Antibodies to latent and replicative EBV antigens such as Epstein–Barr nuclear antigen (EBNA), viral capsid antigen (VCA), and early antigen were found in high titers in RA patients’ sera [24]. In another study, the existence of five anti-EBV antibodies in serum samples from 83 RA patients and 83 HC matched for age, gender, and race was evaluated, individuals with greater preclinical anti-EBV- IgG-EA antibody levels are more likely to develop RA, indicating that EBV reactivation cycles are accelerated during the preclinical phase of RA, the study concluded that both RF and EBV reactivation may play a substantial role in the disease development [25]. *Human Cytomegalovirus* (HCMV) is an inflammatory *Herpesvirus* that remains dormant in its host for life. HCMV has been the subject of various RA hypotheses. There is evidence that HCMV has a dual role in RA, since it is engaged in inflammation and also increases the mRNA-binding protein QKI5, which slows the progression of bone erosion in latently infected RA patients [22]. The reason for this contradictory role is still unknown.

In the early twentieth century, researchers explored the potential association between inadequate dental health and RA, observing improved symptoms of RA in patients with dental health issues after undergoing periodontal infection therapy [26,27]. It becomes apparent that RA progression is influenced by oral microbiota which poor oral health is one of its causing factors [28]. Despite rising evidence on the importance of viruses in the etiology of RA and its therapy, as well as the interplay of viral and bacterial components of the oral microbiome in host [8,29–32], most oral microbiome studies in RA rely on bacterial components of the oral microbiome rather than viral components, creating a gap in our understanding of potential viral implications for the disease.

This present study aims to characterize the composition of oral virome in untreated rheumatoid arthritis patients in comparison to healthy controls at various level. The emphasis lies on aspects such as relative abundance, viral diversity, prevalence and identifying potential discriminative viral biomarkers at species level.

## Materials and methods

### Data collection

In this study, raw DNA sequence data from oral samples were retrieved from the NCBI SRA database (https://www.ncbi.nlm.nih.gov/sra) under BioProject: PRJEB6997 [28]. The inclusion criteria for this study involved untreated RAs matched for age and gender with HCs who exhibited no signs of chronic inflammation. Notably, the study eliminated the influence of age, gender, and RA treatments as confounding factors for the detection of oral viral signatures associated with RA. Supplementary table S1 contains information about the selected samples.

### Bioinformatics and statistical analysis

FastQC v0.11.8 was used to verify the quality of raw DNA-Seq data. Cutadapt v2.8 was used to eliminate adaptor sequences and low-quality bases from raw data. The pre-processed sequencing data were processed using MetaPhlAn3 [33], a tool based on unique clade-specific marker genes identified from a diverse set of reference genomes, encompassing 17,000 entries. This reference set includes 13,500 bacterial and archaeal genomes, 3,500 viral genomes, and 110 eukaryotic taxa. To exclude bacterial, eukaryotic, and archaeal taxa, the functions – ignore bacteria, ignore eukaryotes, and ignore archaea were employed. Taxonomic assignments were made using the internal MetaPhlAn3 database. The feature count table was filtered to eliminate counts >2 with sample prevalence >10%. The final feature count table for downstream analysis was prepared by total-sum scaling (TSS) normalization followed by rarefaction for sample depth normalization. Alpha diversity metrics such as Observed, Shannon, Simpson as well as differential viral communities (beta diversity) between RA and HC groups using the Bray-Curtis and Jaccard index distances based on non-metric multidimensional scaling (NMDS) and the PERMANOVA significance test were calculated in R using the vegan package v2.5.6. Linear discriminant analysis effect size (LEfSe v1.1.01) (LDA score > 2 and *p* < 0.05) [34] was used to detect differentially abundant viral species between RA and HC groups. The receiver operating characteristic analysis (ROC) was used to estimate the predictive value of each discovered viral species. Spearman correlation was used for correlation analysis. Heatmaps of the core virome were created in Microbiome Analyst server.

## Results

**Figure 1.**
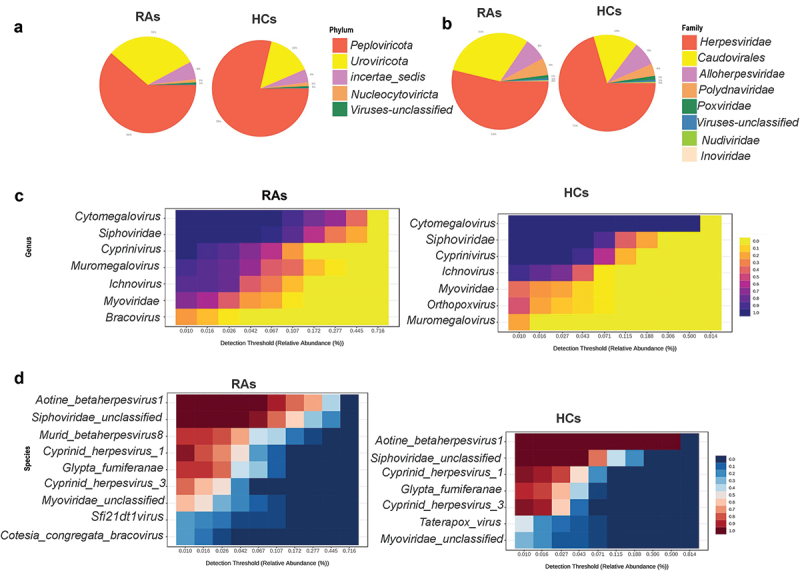
Oral virome composition profiles and core oral virome of RAs and HCs. (a) Pie chart depicting the viral species distribution at the phylum level in RAs and HCs. (b)Pie chart illustrating the family-level distribution of viral species in RAs and HCs (c) Heatmap of the core virome of RAs and HCs at the genus level (d) Heatmap of the core virome of RAs and HCs at the species level.

Initially, raw DNA sequence data for 50 samples were obtained from BioProject PRJEB6997, including 25 untreated rheumatoid arthritis (RA) and 25 matched gender, age healthy controls (HC). Following the exclusion criterion of low viral library size, 45 samples were chosen for downstream analysis, including 21 RAs and 24 HCs. In general, 580,480,711 sequence reads were collected from 45 samples, with a range of 1,806,202 to 37,777,893 and an average of 12,899,571 reads. The total number of DNA viruses from all samples was 29,250,009, with a range between 16,477 and 146,751 and an average of 65,000 viruses per sample. Following a 10% prevalence and reads count 2 > filtration, 101 DNA virus types were recovered and assigned to 11 phyla, 15 families, 25 genera, and 48 species. All samples had achieved a plateau, as indicated by the rarefaction curves. All samples have greater than 99.96% Good’s coverage (Supplementary figure S1). *Peploviricota* and *Uroviricota* are the most common phyla in both groups of RAs and HCs, with *Peploviricota* outnumbering *Uroviricota* (RAs: 61%, HCs: 79%), as demonstrated by an interactive pie chart. A comparison of the two groups revealed that *Peploviricota* is 18% less common in RAs compared to HCs, whereas *Uroviricota* is 14% more prevalent in RAs (Figure 1(a)).

A family level interactive pie chart reveals that *Herpesviridae* and *Caudovirales* are the most abundant families in both groups. Compared to the HCs, the prevalence of *Herpesviridae* is 17% lower in the RAs, while the prevalence of *Caudovirales* is 14% higher (Figure 1(b)). The threshold of prevalence of genus and species in core viromes was set at 20% of samples in each group, with a minimum abundance of 0.01%. The RAs’s core virome included seven genera and nine species taxa, while the HCs core virome had seven genera and seven species taxa. *Siphoviridae, Cytomegalovirus*, and *Cyprinivirus* are the most prevalent in both groups, with 100% prevalence in both. Next, *Ichnovirus* was the most abundant genera in both groups. In RAs, the prevalence rate was 85%, whereas in HCs, it was 91%. *Muromegalovirus* was found in 90% of the RAs and 20% of the HCs, whereas *Myoviridae* was found in 71% of the RAs and 37% of the HCs. *Bracovirus* was only detected in the core virome of RAs with a 23% prevalence, whereas *Orthopoxvirus* was only detected in the core virome of HCs with a 47% prevalence (20% sample prevalence and 0.01% abundance threshold) (Figure 1(c)). Core virome at the species level showed that the most common species are *Siphoviridae unclassified* and *Aotine betaherpesvirus 1*, which have 100% prevalence in each group, *Murid betaherpesvirus 8, Sfi21dt1 virus*, and *Cotesia congregata bracovirus*, which have prevalence percentages of 85%, 23%, and 23%, respectively, and are only found in RAs.

*Cyprinid herpesvirus 1* was found in 95% of both groups, while *Cyprinid herpesvirus 3* was found in 95% of HCs and 76% of RAs. *Myoviridae unclassified* prevalence was 61% in RAs and 29% in HCs. *Taterapox virus* was exclusively detected in HCs and had a 45% prevalence (20% sample prevalence and 0.01% abundance threshold) (Figure 1(d)).

### Oral virome richness and diversity among RAs and HCs

**Figure 2.**
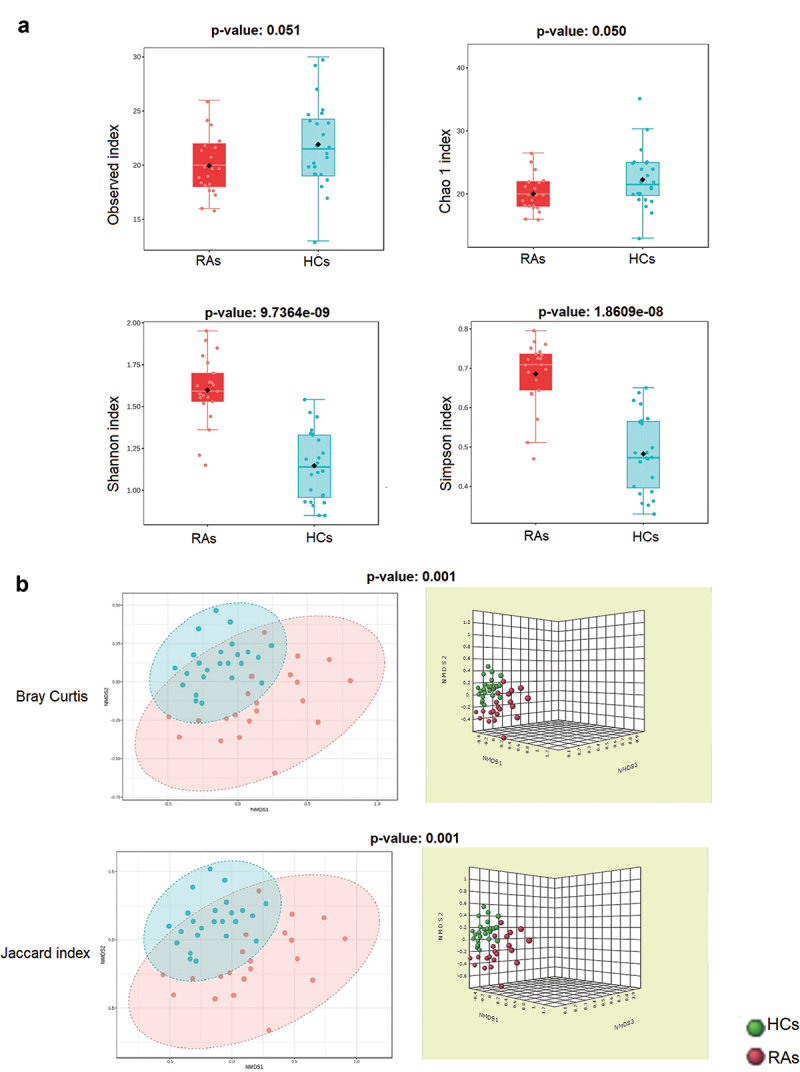
The oral virome richness and diversity and interpersonal variations in RAs and HCs. (a) Boxplot of alpha diversity of observed and Chao1, Shannon and Simpson’s indices reflect the abundance and diversity of species in the samples. (b) PERMANOVA-validated non-metric multidimensional scaling (NMDS) beta variety depicted with Bray-Curtis and Jaccard index distances.

The richness of the oral virome varied considerably between RAs and HCs (alpha diversity: observed index: *p* = 0.051; Chao1 index: *p* = 0.050; Shannon index: *p* < 0.0001; and Simpson’s index: *p* < 0.0001) (Figure 2(a)). The RAs demonstrated greater viral diversity than the HCs group. Bray-Curtis and the Jaccard index distances based on non-metric multidimensional scaling (NMDS) revealed interpersonal differences between RAs and HCs (Bray Curtis and PERMANOVA: *p* < 0. 001; Jaccard index and PERMANOVA: *p* < 0.001) (Figure 2(b)).

### Taxonomic differences of oral virome between RAs and HCs

**Figure 3.**
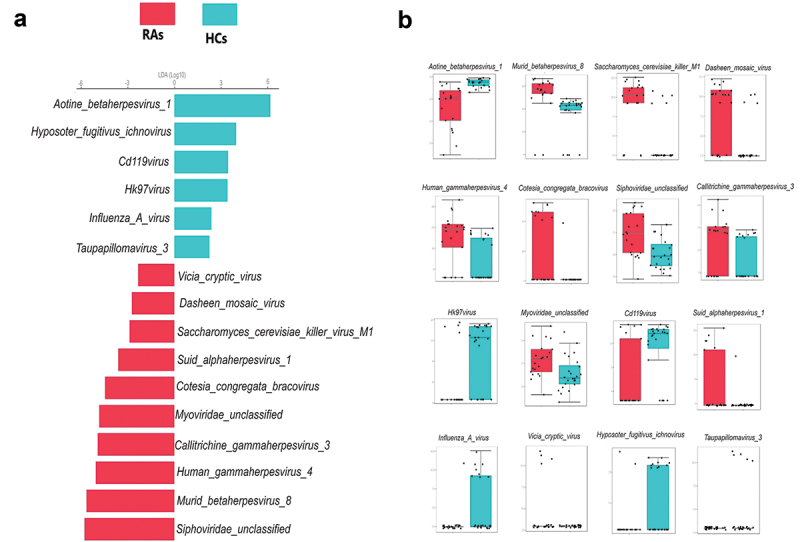
Oral viral signatures significantly different between RAs and HCs. (a) Linear discriminant analysis (LDA) effect size analysis (LEfSe) identified the most differentially abundant viral species between RAs and HCs (*p* < 0.05; LDA score 2). HCs-associated viral species are indicated with negative LDA scores (green), and RAs-associated viral species are indicated with positive LDA scores (red color). (b) pairwise Wilcoxon test for each significant viral signature identified by LEfSe analysis (*p* < 0.05).

LEfSe analysis found that HCs had a greater abundance of *Aotine betaherpesvirus 1, Hyposoter fugitivus ichnovirus, Cd119virus, Hk97virus, Influenza A virus* and *Taupapillomavirus 3*, whereas RAs had a considerably higher abundance of *Aotine betaherpesvirus 1, Siphoviridae unclassified, Murid betaherpesvirus 8, Human gammaherpesvirus 4, Callitrichine gammaherpesvirus 3, Myoviridae unclassified, Cotesia congregata bracovirus, Suid alphaherpesvirus 1, Saccharomyces cerevisiae killer virus M1, Dasheen mosaic virus* and *Vicia cryptic virus* (Figure 3(a)). The pairwise Wilcoxon test of LEfSe analysis for each of the identified viral signature displayed in (Figure 3(b)) (*p* < 0.05).

**Figure 4.**
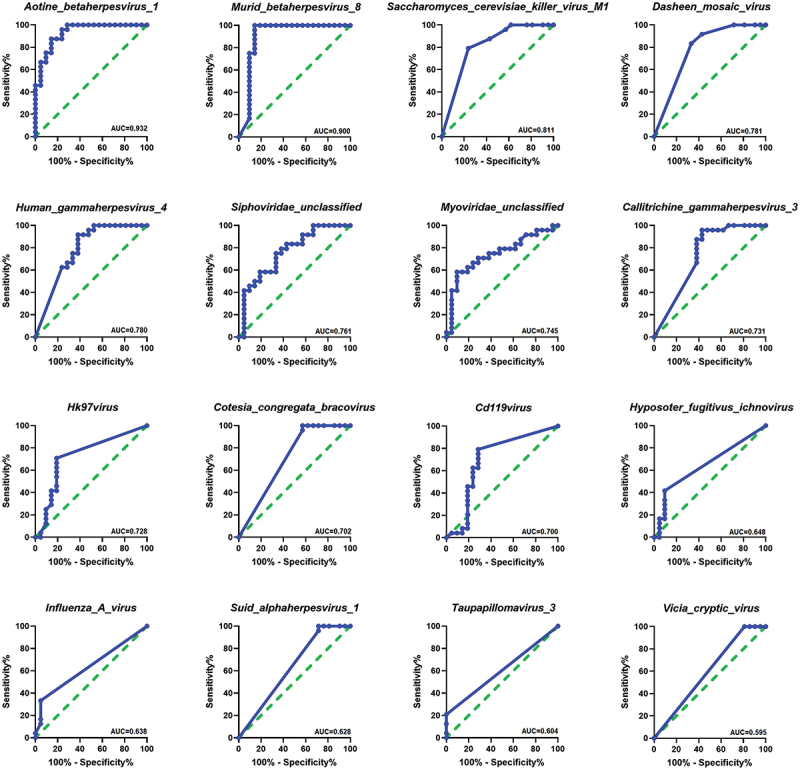
Receiver operating characteristics (ROC) analysis. (a) The area under curves (AUC) values obtained by ROC analysis, show the predictive power of the individual viral signature differs between RAs and HCs,(AUC; 95% CI).

The results obtained from the receiver operating characteristic (ROC) analysis (Figure 4) indicated that these viral signatures such as *Aotine betaherpesvirus 1, Murid betaherpesvirus 8, Saccharomyces cerevisiae killer virus M1, Dasheen mosaic virus, Human gammaherpesvirus 4, Siphoviridae unclassified, Myoviridae unclassified, Callitrichine gammaherpesvirus 3, Hk97virus, Cotesia congregata bracovirus and Cd119virus* provided the area under curve (AUC) value of 0.70 to 0.93 (AUC; 95% CI, *p* < 0.05), whereas the remaining viral signatures such as *Hyposoter fugitivus ichnovirus*, *Influenza A virus, Suid alphaherpesvirus 1, Taupapillomavirus 3* and *Vicia cryptic virus* exhibited AUC values ranging from 0.59 to 0.64 with p-values ranging from 0.083 to 0.084 (AUC; 95% CI) .

## Discussion

Despite evidence suggesting a connection between viruses and the oral microbiome in rheumatoid arthritis (RA), there is a notable absence of comprehensive whole genome sequencing studies on the oral virome at the species level in untreated RA patients. This pilot study seeks to bridge this gap by comparing the oral virome, specifically at the species level, of untreated rheumatoid arthritis patients (RAs) with that of healthy individuals (HCs). The primary objective is to identify specific oral viral signatures associated with RA. A previous study [35] have demonstrated the significance of the oral virome in the pathogenesis of rheumatoid arthritis (RA) offering valuable insights into the intricate connections between the oral virome, encompassing saliva and plaque, and the gut virome among both treated and untreated RA patients, characterizing viral species at the family level using an assembled contigs methodology.

In our current investigation, we deliberately excluded treatment as a potential confounding factor, and we focused on comparing oral virome of untreated RA individuals with healthy controls across various taxonomic levels, including phylum, family, genus, and species level. This comparison was facilitated using the MetaPhlAn pipeline. This study contributes as an initial investigation in identifying discriminative viral biomarkers using LEfSe analysis at the species level associated with RA. The finding showed that several species from *gammaherpesvirus*, *betaherpesvirus*, and *alphaherpesvirus* families were more abundant in RAs. In general, Alpha*-herpesviruses*, such as *Herpes Simplex Virus Type 1 (HSV-1)* and *Herpes Simplex Virus Type 2 (HSV-2)*, establish latent infections in immune system cells, causing acute infections with recurrent inflammation. *Beta-herpesviruses*, like *Cytomegalovirus (CMV)*, contribute to persistent infections, potentially leading to long-term inflammation. *Gamma-herpesviruses*, including *Epstein–Barr Virus (EBV)* and *Kaposi’s Sarcoma-Associated Herpesvirus (KSHV)*, are linked to lymphoproliferative disorders and cancers, inducing chronic inflammation and elevating the risk of malignancies [36,37]. Consistent with this study’s findings, several research have identified a link between *Herpesviruses*, inflammation and RA [22,38–40]. Some evidence connects *Alpha herpesviruses* including *Herpes zoster* and *Herpes simplex viruses* to RA [41,42], *Herpesviruses* have been detected in RA patients’ blood and synovial fluid [40]. It was observed that RA patients receiving glucocorticoids and targeted synthetic DMARDs (Disease-modifying antirheumatic drugs) are at higher risk of *Herpes zoster virus* [42]. In a recent study, it was found that patients with RA exhibited a significantly higher prevalence of HSV II infection (36.34% vs. 24.72%, *p* = 0.015 [43]. *Human gammaherpesvirus 4* or *Epstein Barr virus (EBV)* infection has been demonstrated to be related with RA in adults [23], however the mechanism behind this connection is unknown.

One research study provided evidence that latent *Gammaherpesvirus* infection affects arthritis in mouse models; they suggested that viral latency, and not active virus, is responsible for disease aggravation. Using Age-associated B cells (ABCs) knockout mice, the researchers demonstrated for the first time that ABCs are mechanistically required for viral enhancement of RA, meaning that latent *gammaherpesvirus* infection impacts ABCs and causes arthritis and the murine analogue of EBV, *Gammaherpesvirus 68* (γHV68) generated more severe collagen-induced arthritis and a Th1-skewed immune profile comparable to human illness [44]. In this present study, *Human gammaherpesvirus 4* and *Callitrichine gammaherpesvirus 3* were found to be abundant in the oral cavity of RAs for the first time; this result supports future exploration of these analogues with oral cavity origin in disease enhancement.

*Betaherpesvirus*, particularly *Cytomegalovirus* which generates inflammation and remains latent in its host for life, has been at the center of various hypotheses for RA and plays dual roles in RA pathogenesis, including bone erosion prevention and inflammation [22,44]. *Murid betaherpesvirus 8*, a common rat commensal virus, was shown to be enriched in human RA in this study, *Aotine betaherpesvirus 1* from *Cytomegalovirus* was more enriched in HCs than RAs. Previous studies showed that *Cytomegalovirus* reduces bone erosion by activating the mRNA-binding protein QKI5, thereby decreasing the rate of bone erosion in patients with latent RA infection. [^22,45]^ However, the evidence indicated that the inflammatory role of *Betaherpesvirus*, specifically *Cytomegalovirus*, may be a confounding factor in associations including periodontitis. The negative correlation between *Cytomegalovirus* and *Muromegalovirus* (r: −0.6228, *p* < 0.0001) in this study’s entire cohort suggests that additional research is necessary to determine whether *Cytomegalovirus* and *Muromegalovirus* are friends or foes of RA caused by the *Betaherpesvirus* subfamily (Supplementary figure S2).

In addition, the Sac*charomyces cerevisiae killer virus M1* (ScV-M1) was enriched in RAs. As a virulent virus, ScV-M1 is known for its aggressive impact on *Saccharomyces cerevisiae*, particularly through the production of a unique virulent toxin, K1 whose specific characteristics include its potential mode of action, molecular structure, and immunomodulatory effects [46]. Recently, it was discovered that RA patients had a high prevalence of anti-Saccharomyces cerevisiae antibodies (ASCA) IgG and IgA antibodies. [^47]^ More research is warranted to determine how the virus contributes to the pathogenesis of RA.

*Hk97virus* a member of the *Siphoviridae* family and *Cd119virus* and a member of the *Myoviridae* family were enriched in HCs. These bacteriophages play a pivotal role in the bacterial ecosystem. These viruses are specific in their targeting, often infecting particular bacterial strains. Upon infection, these bacteriophages undergo a reproductive cycle within the bacterial host, ultimately leading to the lysis or destruction of the host bacterium. Their role extends to controlling bacterial populations, and they are actively studied for their potential applications in phage therapy, a novel approach to combat bacterial infections [48,49]. However, no research has shown a link between these phages and RA as of yet. Nevertheless, these phages are necessary for maintaining the balance of oral bacteria and possess antibacterial properties that could serve as a bacteriophage therapy for bacterial-induced inflammation. A Spearman correlation heatmap was utilized in the study to examine the potential correlation between the oral virome in RA. The investigation found that there were both positive and negative associations between the detectable viral signatures of RAs and HCs. These results suggest a possible complex interaction between RA and the oral virome (Supplementary figure S3). Future studies may translate this collaboration into common metabolic processes by which these viruses can activate the immune system and induce inflammation in RAs or protect against the development of rheumatoid arthritis in HCs. This study has some limitations due to the use of SRA data, which gave little information on the clinical characteristics of the submitted samples that could be considered confounders and the relatively small sample size. Some samples were omitted from the analysis because, as mentioned, the size of the viral library was quite small. The reason for this is that the project study from which the raw data were collected was intended to examine the bacterial population. It is proposed that, for future research, an optimal viral DNA extraction be done to stabilize the viral species in the samples. The study of the bacteriome and the virome simultaneously, as well as their interaction, will give light on the mechanistic relationship between RA and the oral microbiome.

Although viruses primarily affect the immune system in latency, monitoring cohort of RAs before and after immunomodulation will provide a better understanding of the process by which latent infection stimulates the immune system.

While this study specifically focused on DNA viruses, it is essential to recognize the role of RNA viruses in the complex dynamics of diseases such as RA. The exclusion of RNA viruses in this pilot study highlights a limitation in the current scope. Future investigations should aim to integrate both RNA and DNA sequencing techniques to obtain a more comprehensive and nuanced understanding of the complete virome. By simultaneously profiling DNA and RNA, researchers can explore a broader range of viral components, providing a more holistic view of the oral microbiome and its potential implications for the pathogenesis of RA.

Despite these valuable insights, we acknowledge certain limitations in our study. We recognize the need for more detailed quantitative analyses. Specific areas, such as viral load, replication dynamics, lysogeny, lytic activity, genome completeness, and other functional characteristics, warrant further exploration. One other limitation of our study is the absence of determination of participants’ oral health status, which may impact the identification of viral biomarkers associated with health conditions such as periodontitis and caries. Given the limited research on oral virome profiles in healthy individuals compared to those with periodontitis or caries, future studies should address this aspect to ensure a comprehensive understanding of the oral virome composition in rheumatoid arthritis and healthy controls.

## Conclusion

In conclusion, our study contributes to the initial explorations of the oral virome in individuals with untreated rheumatoid arthritis compared to healthy controls without RA inflammation. This comprehensive characterization spans various taxonomic levels, including phylum, family, genus, and species. Notably, our findings suggest a potential oral origin for *Herpesviruses* previously identified in the blood and synovial fluid of RA patients.

In addition, our investigation aims to pinpoint specific oral viral biomarkers identified through LEfSe analysis that are associated with rheumatoid arthritis within this cohort. Notably, biomarkers highlighted in the LEfSe analysis include *Aotine betaherpesvirus 1, Siphoviridae unclassified, Murid betaherpesvirus 8, Human gammaherpesvirus 4, Callitrichine gammaherpesvirus 3, Myoviridae unclassified, Cotesia congregata bracovirus, Suid alphaherpesvirus 1, Saccharomyces cerevisiae killer virus M1, Dasheen mosaic virus*, and *Vicia cryptic virus*. These biomarkers exhibit differential abundances between healthy controls and rheumatoid arthritis patients and have the potential to serve as crucial indicators for the early diagnosis of rheumatoid arthritis, representing a noteworthy advancement in the nascent stages of research on the oral virome in the context of this complex autoimmune condition.

## Supplementary Material

Supplemental Material

supplementary figure 1.tif

supplementary figure 2..tif

Supplementary figure 3.jpg

## Abbreviations

ACPAs: Anti-citrullinated peptide antibodies
AUR: Area under curve
bDMARD: biological disease-modifying antirheumatic drugs
EBNA: Epstein-Barr nuclear antigen
EBV: *Epstein-Barr virus*
γHV68: *Gammaherpesvirus 68*
HCMV: *Human Cytomegalovirus*
LEfSe: Linear discriminant analysis Effect Size
NMDS: Non-metric multidimensional scaling
ROC: Receiver operating characteristic
RA: Rheumatoid arthritis
RF: Rheumatoid factor
ScV-M1: *Saccharomyces cerevisiae killer virus M1*
TSS: Total Sum Scaling
TFNI: Tumor necrosis factor inhibitor
VCA: Viral capsid antigen
WGS: Whole genome sequence
