# Supplementary material for: Distinct oral DNA viral signatures in rheumatoid arthritis: a Pilot study
Source: J Oral Microbiol. 2024 May 1;16(1):2348260. doi: 10.1080/20002297.2024.2348260 (PMC11064737; doi:10.1080/20002297.2024.2348260)
Supplement: Supplemental Material [file ZJOM_A_2348260_SM1746.doc]

| **SRA id** | **Sample id** | **Gender** | **Age** | **Instrument Model** | **Read Count** | **Base Count** |
| --- | --- | --- | --- | --- | --- | --- |
| ERR589382 | RAH10 | Female | 68 | Illumina HiSeq 2000 | 13288310 | 2611287993 |
| ERR589386 | RAH13 | Female | 46 | Illumina HiSeq 2000 | 14749622 | 2901260486 |
| ERR589392 | RAH18 | Female | 41 | Illumina HiSeq 2000 | 9935733 | 1945499057 |
| ERR589394 | RAH19 | Female | 37 | Illumina HiSeq 2000 | 6668606 | 1309438066 |
| ERR589396 | RAH24 | Female | 53 | Illumina HiSeq 2000 | 14748505 | 2889024250 |
| ERR589400 | RAH27 | Female | 48 | Illumina HiSeq 2000 | 2740751 | 538105390 |
| ERR589402 | RAH29 | Female | 42 | Illumina HiSeq 2000 | 4084729 | 803086012 |
| ERR589404 | RAH31 | Female | 43 | Illumina HiSeq 2000 | 6612380 | 1297108723 |
| ERR589406 | RAH32 | Female | 41 | Illumina HiSeq 2000 | 16038837 | 3147227814 |
| ERR589408 | RAH40 | Female | 40 | Illumina HiSeq 2000 | 20994792 | 4094194340 |
| ERR589410 | RAH43 | Female | 46 | Illumina HiSeq 2000 | 12459304 | 2445283716 |
| ERR589412 | RAH47 | Female | 58 | Illumina HiSeq 2000 | 11294910 | 2215547514 |
| ERR589414 | RAH50 | Female | 46 | Illumina HiSeq 2000 | 12247952 | 2391740131 |
| ERR589416 | RAH51 | Female | 44 | Illumina HiSeq 2000 | 4538283 | 890752677 |
| ERR589422 | RAH62 | Female | 23 | Illumina HiSeq 2000 | 12413640 | 2429572963 |
| ERR589426 | RAH7 | Female | 54 | Illumina HiSeq 2000 | 30348547 | 5963714299 |
| ERR589430 | RAH71 | Female | 48 | Illumina HiSeq 2000 | 19228398 | 3765604526 |
| ERR589436 | RAH93 | Female | 57 | Illumina HiSeq 2000 | 6449347 | 1256287140 |
| ERR589465 | RA50 | Female | 50 | Illumina HiSeq 2000 | 22769917 | 4459791113 |
| ERR589466 | RA79 | Female | 36 | Illumina HiSeq 2000 | 12145044 | 2382235611 |
| ERR589486 | RA113 | Female | 46 | Illumina HiSeq 2000 | 6055525 | 1189482760 |
| ERR589487 | RA118 | Female | 59 | Illumina HiSeq 2000 | 14133197 | 2773274991 |
| ERR589488 | RA135 | Female | 52 | Illumina HiSeq 2000 | 37777893 | 7439972553 |
| ERR589489 | RA138 | Female | 67 | Illumina HiSeq 2000 | 19693993 | 3866189983 |
| ERR589491 | RA182 | Female | 49 | Illumina HiSeq 2000 | 23013142 | 4550318955 |
| ERR589492 | RA51 | Female | 57 | Illumina HiSeq 2000 | 18036957 | 3543062227 |
| ERR589493 | RA54 | Female | 44 | Illumina HiSeq 2000 | 17527192 | 3418299311 |
| ERR589494 | RA001 | Female | 32 | Illumina HiSeq 2000 | 2816717 | 548446056 |
| ERR589495 | RA155 | Female | 40 | Illumina HiSeq 2000 | 22090479 | 4295016924 |
| ERR589497 | RA122 | Female | 62 | Illumina HiSeq 2000 | 13614046 | 2658796575 |
| ERR589519 | RA150 | Female | 49 | Illumina HiSeq 2000 | 7802900 | 1519868797 |
| ERR589520 | RA157 | Female | 55 | Illumina HiSeq 2000 | 4159838 | 814505017 |
| ERR589521 | RA197 | Female | 34 | Illumina HiSeq 2000 | 10644489 | 2065151713 |
| ERR589522 | RA202 | Female | 53 | Illumina HiSeq 2000 | 15859900 | 3083174293 |
| ERR589523 | RA209 | Female | 34 | Illumina HiSeq 2000 | 4301102 | 840599324 |
| ERR589524 | RA225 | Female | 46 | Illumina HiSeq 2000 | 24731088 | 4770257650 |
| ERR589525 | RA240 | Female | 56 | Illumina HiSeq 2000 | 23936937 | 4628105482 |
| ERR589384 | RAH12 | Male | 52 | Illumina HiSeq 2000 | 8779581 | 1710151588 |
| ERR589390 | RAH15 | Male | 46 | Illumina HiSeq 2000 | 5328126 | 1041456829 |
| ERR589418 | RAH53 | Male | 45 | Illumina HiSeq 2000 | 6471609 | 1273562556 |
| ERR589424 | RAH68 | Male | 38 | Illumina HiSeq 2000 | 4336025 | 854793226 |
| ERR589428 | RAH70 | Male | 48 | Illumina HiSeq 2000 | 8538556 | 1681139931 |
| ERR589434 | RAH89 | Male | 42 | Illumina HiSeq 2000 | 1806202 | 357390754 |
| ERR589490 | RA179 | Male | 47 | Illumina HiSeq 2000 | 15549248 | 3056934493 |
| ERR589496 | RA158 | Male | 60 | Illumina HiSeq 2000 | 9718362 | 1913423647 |
