# Supplementary figures and images for: Distinct oral DNA viral signatures in rheumatoid arthritis: a Pilot study
Source: J Oral Microbiol. 2024 May 1;16(1):2348260. doi: 10.1080/20002297.2024.2348260 (PMC11064737; doi:10.1080/20002297.2024.2348260)

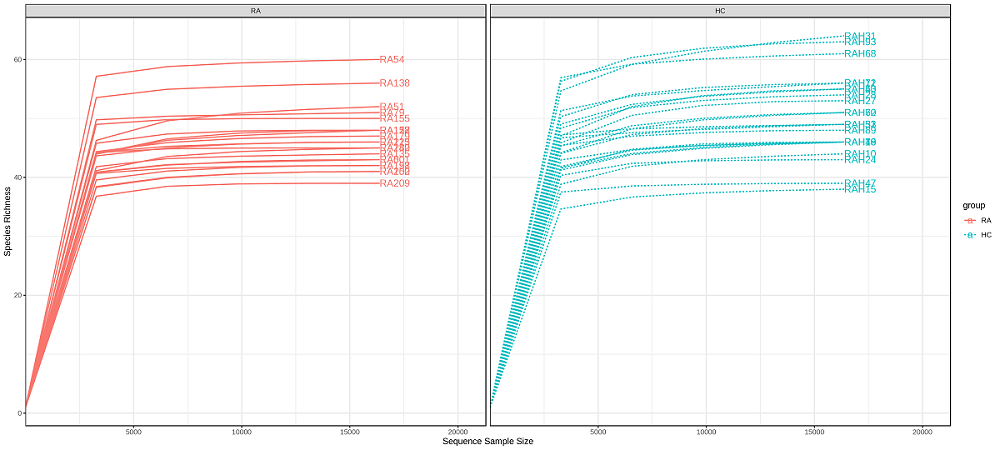

Supplement: supplementary figure 1.tif [file ZJOM_A_2348260_SM3086.tif]

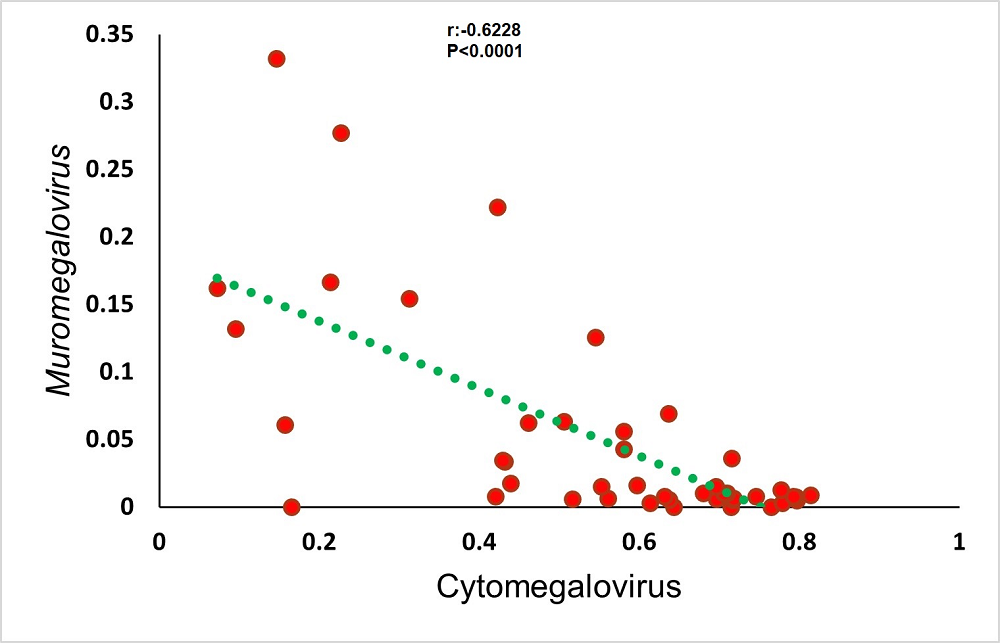

Supplement: supplementary figure 2..tif [file ZJOM_A_2348260_SM3085.tif]

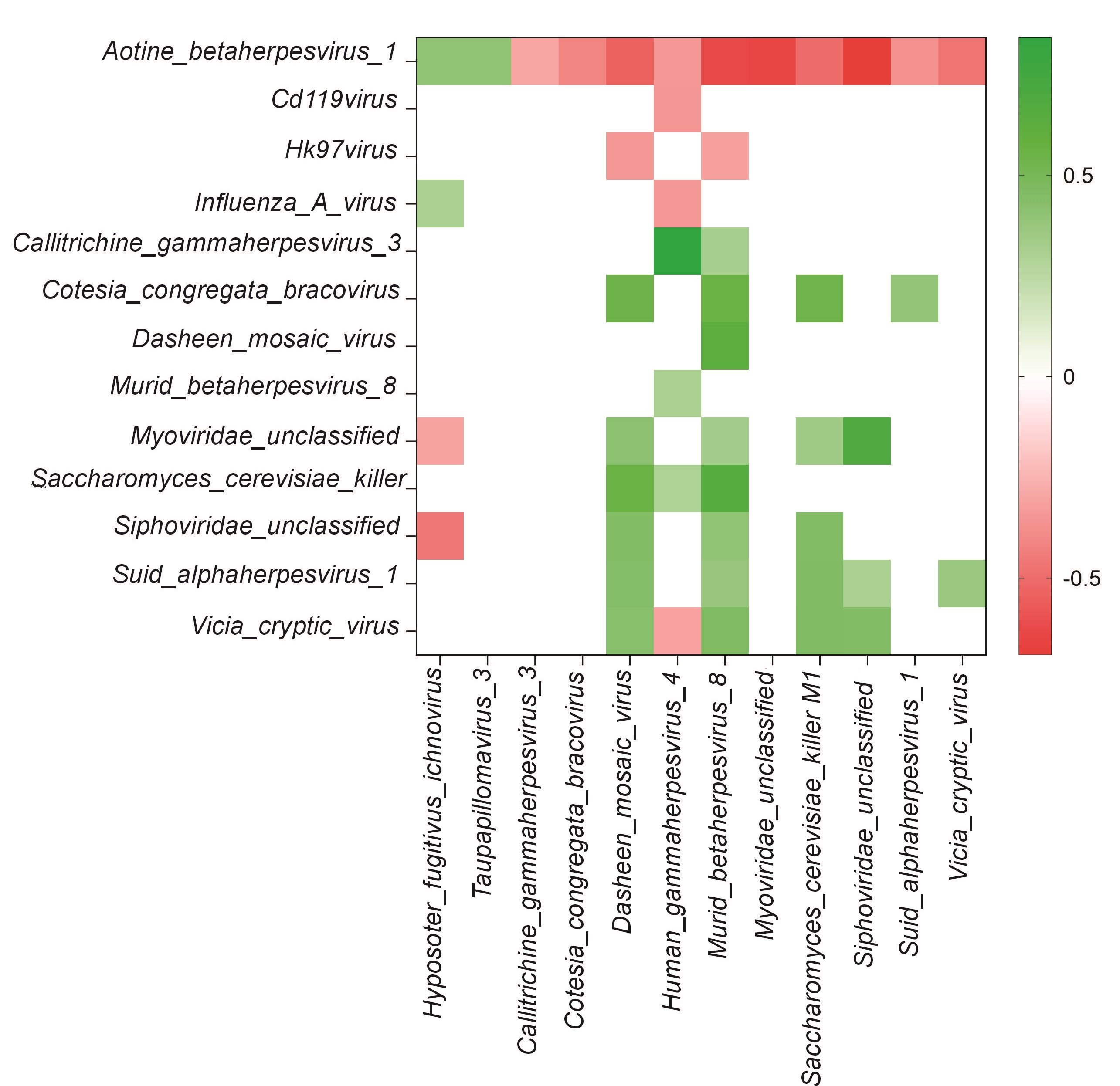

Supplement: Supplementary figure 3.jpg [file ZJOM_A_2348260_SM3084.jpg]
